# VEXAS Syndrome: A Novelty in MDS Landscape

**DOI:** 10.3390/diagnostics12071590

**Published:** 2022-06-29

**Authors:** Marie Templé, Olivier Kosmider

**Affiliations:** 1 Cochin Hospital, Université de Paris, F-75006 Paris, France; marie.temple@aphp.fr; 2 Institut Cochin, Université de Paris Cité, CNRS UMR8104, INSERM U1016, F-75014 Paris, France

**Keywords:** autoinflammatory disease, VEXAS (vacuoles, E1 enzyme, X-linked, autoinflammatory, somatic) syndrome, myelodysplastic syndrome

## Abstract

Fever, inflammation and vacuoles in hematopoietic cells represent the main features associated with VEXAS syndrome, a new prototype of autoinflammatory disorders genetically characterized by somatic mutation of the *UBA1* gene which encodes the enzyme1-activating enzyme (E1) required for ubiquitin signaling. Described very recently, patients with VEXAS syndrome present a systemic autoinflammatory syndrome associated with hematological impairments, especially cytopenias whose pathophysiology is mainly non-elucidated. Initially diagnosed in elderly male patients, VEXAS syndrome was frequently associated with a diagnosis of myelodysplastic syndromes (MDS) leading the medical community to first consider VEXAS syndrome as a new subtype of MDS. However, since the first description of VEXAS patients in 2021, it appears from the multitude of case reports that MDS associated with VEXAS are different from the classically described MDS.

## 1. Introduction

VEXAS syndrome (acronym for vacuoles, E1 enzyme, X-linked, autoinflammatory, somatic) is a monogenic disease described by David Beck and colleagues from the National Institutes of Health (NIH) in December 2020. VEXAS patients have a systemic autoinflammatory disease associated with hematological impairments and disorders, mainly myelodysplastic syndrome (MDS) [1]. The description of VEXAS syndrome is new evidence of a link between MDS and systemic autoimmune and/or inflammatory diseases (AID) already reported in the literature [2]. Since then, the report of many VEXAS cases has improved the clinico-biological description of this new syndrome and suggests an underestimated prevalence of the disease, as shown by the French multicenter cohort of 116 VEXAS patients [3]. It also appears that VEXAS MDS seem to be different from the more classical MDS in terms of both heterogeneity and molecular landscape.

## 2. VEXAS Syndrome

Using an original genotype-first approach of selecting patients initially on the basis of gene rather than phenotype similarities, Beck and his team describe a cohort of 25 male patients with a somatic mutation in the ubiquitin-like modifier activating the enzyme 1 (UBA1) gene involved in the protein ubiquitylation system. This discovery was obtained by screening the exome and genome results analyses of 2560 individuals: 1477 patients listed for undiagnosed recurrent fevers and/or systemic inflammation and 1083 patients with unclassifiable atypical disorders. Three patients were identified with a somatic mutation at codon 41 of the UBA1 gene (p.Met41) located on the X chromosome. These mutations were predicted to be deleterious in silico and were not reported in public databases. A second, more targeted, screening of patients with clinical signs similar to the three patients allowed the establishment of a cohort of these 25 VEXAS patients. The 25 patients in the original cohort are exclusively male, so it was suggested that the female second X-chromosome allele protects against the detrimental action of the mutated UBA1 allele [1]. The more recent description of several VEXAS women with karyotypic X monosomy supports this hypothesis [4,5,6]. The true prevalence of this syndrome is unknown to date, but various descriptions tend to show that this entity is not so rare with, however, a majority of those affected being male.

Like diseases with alterations of the ubiquitylation and proteasome system [7], the clinico-biological presentation of this VEXAS syndrome is very heterogeneous. Typically, VEXAS patients present a systemic inflammatory disease with unexplained episodes of fever, involvement of the lungs, skin, blood vessels and joints. The majority of patients present the characteristic clinical signs of inflammatory diseases such as Sweet’s syndrome, polyarteritis nodosa and recurrent polychondritis. From a hematological point of view, in the initial NIH cohort VEXAS patients develop cytopenias and notably macrocytic anemia (96%), myelodysplastic syndrome (24%), multiple myeloma (20%) and thromboembolic disorders (44%) [1].

**Figure 1:**
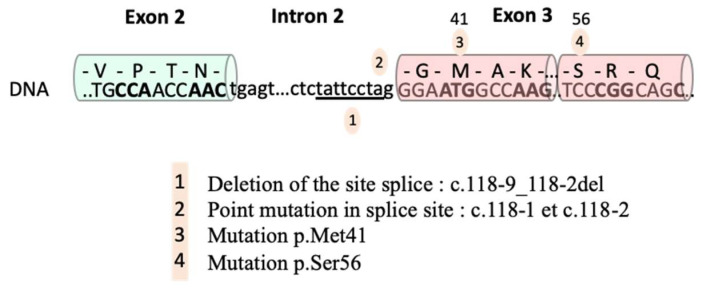
Mutations in the UBA1 gene associated with VEXAS syndrome. According to Templé et al. [9].

Molecular diagnosis of VEXAS syndrome is made by the sequencing of the UBA1 gene. The 3 most frequent mutations affect methionine 41 of exon 3 of UBA1: p.M41T (c.122T>C), p.M41V (c.121A>G) and p.M41L (c.121A>C). Since then other mutations have been reported, such as Splice region mutations at exon 3 (c.118-2A>C and c.118-1G>C) [8] and (c.118- 9_118-2del) [9] as well as a mutation affecting codon 56 (c.167C>T) in a patient with a less marked clinical syndrome, have also been reported [10]. All these mutations are shown in Figure 1.

The clinico-biological and generic description of the VEXAS syndrome has been refined thanks to numerous case reports, such as results of a French multicenter cohort describing 116 VEXAS patients in a retrospective study [3]. VEXAS syndrome is mostly described in men with a progressive onset of the disease after 50 years of age. The clinical manifestations found in these 116 patients are similar to those reported in the initial study but with a higher rate of association with MDS (50%). The distribution of the type of UBA1 mutations is also similar to that reported in the literature, with the order of frequency being p.M41T (44.8%), p.M41V (30.2%), p.M41L (18.1%) and splice mutations (6.9%). An unsupervised hierarchical analysis allowed the division of these patients into three main phenotypically distinct groups based on the integration of clinical and biological data. Survival data affiliated with these three groups show that VEXAS patients with MDS have a poorer survival rate compared with other patients and that the p.M41L mutation appears to be associated with milder disease, suggesting a potential phenotype–genotype association in VEXAS.

Thrombotic complications are reported in approximately 40% of VEXAS patients [1], with a predominance of venous (36.4%) rather than arterial (1.6%) thromboembolic disorders [11]. High rates of thrombosis following autoinflammatory diseases or immune dysregulation have been described in the literature [12]. In VEXAS patients, it is assumed that the ubiquitylation defect leads to a deregulation of innate immunity and a systemic inflammation favoring the occurrence of thrombus [11].

**Figure 2:**
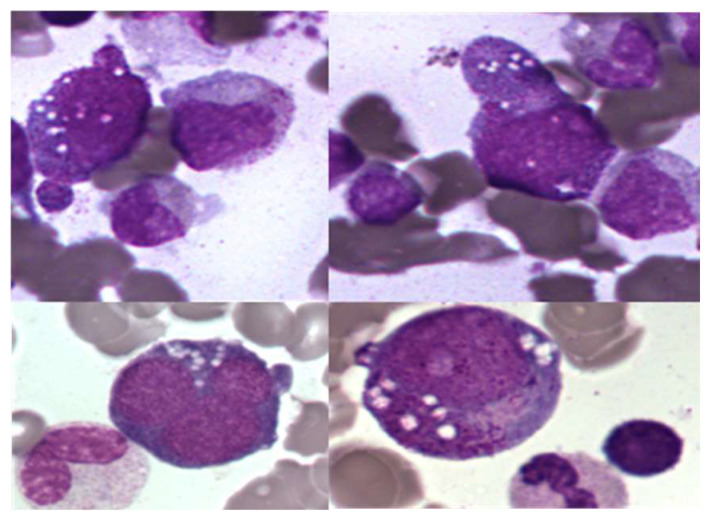
Cytoplasmic vacuolation of granular and erythroid progenitor cells, ×100.

Examination of bone marrow smears from VEXAS patients shows the presence of vacuoles in the cytoplasm of myeloid medullary progenitor cells, predominantly in granular and erythroid precursors (Figure 2, personal photos, MGG staining). They do not appear to be found in the cytoplasm of mature circulating blood cells, and their exact composition is not yet defined. These vacuoles are of course not pathognomonic of the VEXAS syndrome since they can also be described, for example, during sepsis, chronic alcoholism, zinc excess or copper deficiency. A biological score has been proposed to guide the cytologist: the presence of medullary cytoplasmic vacuoles (positive threshold set at >1 vacuole per cell) in more than 10% of neutrophil precursors associated with a suggestive clinical context should prompt a search for a UBA1 gene mutation by sequencing [13]. The number of vacuoles is heterogeneous between patients. The absence of vacuole should not be a criterion for exclusion of VEXAS diagnosis since some patients do not show vacuole on a bone marrow smear [9,14].

From a functional point of view, the UBA1 gene codes for the enzyme UBA1 or Enzyme E1, are involved in the initiation of the protein ubiquitylation process. This post-translational modification is essential in the regulation of protein turnover, especially those involved in the cell cycle, cell death, signal transduction, etc., by allowing them to be sent to the proteasome, a protein degradation organelle. Ubiquitylation is also involved in non-proteolytic functions: assembly of multiprotein complexes, intracellular signaling, inflammatory signaling, DNA repair, autophagy process, etc. [15]. Given its pleiotropic function, it is expected that functional alteration of E1 causes clinical heterogeneity in VEXAS patients. The binding of ubiquitin to lysine residues of target proteins is enabled by the sequential action of three enzymes: E1 (ubiquitin activating enzyme), E2 (conjugating enzyme) and E3 (ligase enzyme). In contrast to the E1 enzyme, which is mainly encoded by UBA1, there are dozens of distinct E2 conjugating enzymes and hundreds of E3 ligase enzymes that ensure the specificity of ubiquitylation. E1 forms a thioester bond with the terminal region of ubiquitin through an ATP-dependent mechanism. The ubiquitin is then transferred to a sulfide group of a conjugating enzyme E2. Finally, E3 binds the ubiquitin molecule to a lysine of the target protein. The anchoring of at least four ubiquitin molecules to the target protein is necessary for it to address the proteasome. Different models of ubiquitylation exist (mono-ubiquitylation, multi-ubiquitylation or poly-ubiquitylation) and are distinguished according to the number of ubiquitin binding sites on the protein to be degraded and the type of binding (linear or polymeric) [15].

Physiologically, UBA1 encodes two protein isoforms: UBA1a for nuclear localization, translated at methionine 1 (exon 2), and UBA1b for cytoplasmic localization, a shorter isoform since its translation is initiated at methionine 41 (M41) (exon 3) [1]. Beck et al. have shown by a functional study that the mutation in M41 leads to a shift in the reading frame and to the synthesis of a pathological isoform UBA1c presenting a catalytic deficiency disrupting the whole ubiquitylation process. Interestingly, the UBA1 clone is detected in hematopoietic progenitor cells and in circulating myeloid cells and is absent from mature lymphoid cells indicating clonal heterogeneity [1]. As previously mentioned, other mutations have been reported including a mutation at amino acid 56 (c.167C>T) in a patient with a less marked clinical syndrome. In this case, the UBA1 clone is restricted to the myeloid compartment and does not result in the synthesis of a pathological UBA1c isoform. However, it demonstrates a temperature-dependent catalytic deficiency of the E1 [10].

As initially presented, VEXAS patients show an increased level of serum inflammatory markers (tumor necrosis factor, interleukin-8, interleukin-6, interferon-inducible protein-10, interferon-gamma, C-reactive protein) and aberrant activation of innate immune signaling pathways [1]. Loss of UBA1b validated by a Zebra-fish model causes a biological inflammatory phenotype similar to the one found in VEXAS patients [1]. The large number of case reports has improved the clinico-biological description of this new syndrome, but more functional studies are now needed to understand the pathophysiological mechanism of this disease combining inflammation and hematological disorders in its globality. Preliminary results show the possible involvement of non-specific inflammatory pathways such as IFNalpha and IFNbeta, and cellular stress, such as the unfolded protein response (UPR) pathway linked to the endoplasmic reticulum [1].

VEXAS is a syndrome resistant to the classical therapeutic arsenal and is depicted as a disease with a poor prognosis and requiring the use of high-dose glucocorticoids. To date, the treatment data available are retrospective and involve the use of different therapeutic approaches, often numerous, depending on the clinicians involved in the management. Indeed, 40% of the patients in the initial publication died of disease-related causes or secondary to treatment toxicity, suggesting the importance of finding an effective therapy [1]. A team from Lyon retrospectively evaluated the efficacy of different lines of treatment in 11 VEXAS patients who had received a median of three lines of treatment, with the time to initiation of the next treatment as an efficacy criterion [8]. They also show encouraging results for treatment with azacitidine, a demethylating agent currently used in the treatment of MDS. Its efficacy is also reported by another team, which reports a response in 46% (cohort of 11 Vexas patients), with the efficacy criterion being a reduction in corticosteroid doses [16]. Similarly, a response to azacitidine was reported in two VEXAS patients, both with a DNMT3A mutation, while one VEXAS patient with a TET2 mutation did not respond to treatment, suggesting an increased sensitivity to azaciditine in VEXAS patients with a DNMT3A mutation [17]. The results of these studies need to be confirmed with larger cohorts and longer follow-up. It should be noted that one patient presented an indolent form of the disease without recourse to specific treatment over a long period (87 months) [8].

The use of inhibitors of the Janus kinase pathway appears to be a promising therapeutic avenue as shown by the results of a multicenter, retrospective analysis of a cohort of 30 patients who received JAK inhibitors. Ruxolitinib was found to be more effective than other JAK inhibitors: a significant increase in hemoglobin and platelet levels in patients treated with ruxolitinib compared to other patients treated with JAK inhibitors was reported, as well as a reduction in corticosteroid dependence in patients treated with JAK inhibitors after 6 months of treatment. One hypothesis raised is that the gain in efficacy of ruxolitinib compared to other inhibitors is related to the target specificity of ruxolitinib, with an inhibitory action essentially of JAK1, JAK2. However, these treatments do not allow the eradication of the UBA1 clone, since the UBA1 clone is still visible by sequencing on the follow-up samples [18].

The evaluation of the efficacy of allogeneic bone marrow transplantation in VEXAS syndrome is described as promising but will require a strict evaluation of the benefit/risk balance for the patient due to the high mortality of this therapeutic option. A phase II trial is currently being set up to evaluate the efficacy of allogeneic hematopoietic stem cell transplantation in a cohort of VEXAS patients [19]. Actually, this is the only possibility of a priori curative treatment of this syndrome.

To mitigate the poor prognosis of VEXAS patients linked to a limited therapeutic arsenal, it will be important that these patients benefit from early diagnosis, multidisciplinary management (rheumatologists, clinical hematologists and biologists) and regular clinical and molecular follow-up.

VEXAS syndrome is a deregulation of the ubiquitylation system leading to excessive deregulation of inflammation pathways and induction of generalized stress. Beck points out that this inflammatory pathology is superimposed on other diseases of the ubiquitin-proteasome system such as proteasome-associated autoinflammatory syndrome known to have excessive deregulation of immunity. In a detailed review of diseases involving deregulation of the ubiquitin–proteasome system, Beck suggests that the development of substrate-specific activator molecules, such as UBA1 in VEXAS syndrome or targeting the proteasome, could be beneficial in the management of these complex diseases. They also propose that one solution may be to inhibit cellular stress pathways such as the UPR pathway that are frequently activated in IAD [7]. The disappearance of the UBA1 clone at molecular sequencing after the introduction of a new therapeutic line would indeed be a good argument for the effectiveness of the treatment [18].

## 3. VEXAS and Myelodysplastic Syndrome

It is described that approximately 50% of VEXAS patients have MDS [3]. The association between MDS and AID is already described in the literature [20], with VEXAS syndrome providing further genetic evidence. Interestingly, the available data suggest that VEXAS MDS differ from “classical” MDS. To understand this difference in the MDS of the VEXAS patient, it is important to note the existing heterogeneity within the “classic” MDS in terms of their phenotype, prognosis, molecular landscape and therapeutic management.

Briefly, MDS are acquired heterogeneous hemopathies caused in particular by clonal alterations of hematopoietic stem cells (HSC) responsible for inefficient hematopoiesis. Inefficient hematopoiesis is responsible for signs of bone marrow dysplasia, cytopenias and an increased risk of transformation into acute myeloid leukemia (AML) [21]. MDS are relatively common diseases of the elderly (incidence 3/100,000 population), preferentially affecting men in their 70s [22]. MDS cases can be classified as de novo and therapy-related MDS (10–20%) secondary following treatment with chemotherapy, radiotherapy or exposure to various toxic substances (benzene, hydrocarbons, pesticides, etc.). More recently, germline-predisposed MDS have been described, the incidence of which is not yet fully elucidated [23]. The main complications of MDS are related to the depth of cytopenias (asthenia, hemorrhages, infections) as well as to the potential transformation into AML occurring in 30% of cases [24].

In the last 10 years, studies have also shown the role of the deregulated marrow microenvironment with immune dysregulation in the genesis of MDS [25]. There are two non-exclusive schemes to depict the pathophysiology of MDS. The first, and most approved, proposes that emerging clonal HSCs modify the marrow microenvironment. This reprogramming of the hematopoietic niche contributes to the survival advantage of clonal HSC and leads to the progressive suppression of normal hematopoiesis. The second scheme considers the alteration of the microenvironment as the initiating event of MDS. This would create a genetic instability responsible for the selection and clonal expansion of HSCs. Different studies have shown the reciprocal communication between the two compartments [26].

The diagnostic criteria for MDS are those of the World Health Organization (WHO) 2016. Thus, it is necessary to perform a myelogram coupled with a blood sample for cytological, cytogenetic and molecular evaluation [24]. The current WHO classification also incorporates cytogenetic data: MDS with deletion of the long arm of chromosome 5 (del5q) on karyotype is a distinct subgroup of MDS. The diagnostic criteria for MDS are those of WHO 2016 and are based on cytological evaluation of blood and marrow, cytogenetic data (case of deletion of the long arm of chromosome 5) and in some cases detection of molecular abnormalities [27].

In 90% of cases, MDS patients have gene mutations affecting genes from different classes: epigenetic regulators (DNA methylation or chromatin compaction such as TET2, ASXL1, DNMT3A), spliceosome genes (SF3B1, SRSF2, ZRSR2), transcription factors (TP53, RUNX1, ETV6), genes involved in signaling pathways (N/KRAS, JAK2, CBL), or in the cohesin complex (STAG2, RAD21) [28]. The acquisition of an initiating mutation and additional mutations gives the host cell a selective advantage (proliferation, survival) and allows its clonal expansion at the expense of normal HSCs. Thus, the existence of HSC subclones contributes to the intra-tumor heterogeneity classically described in MDS.

There is heterogeneity in the prognosis of MDS. Tools for stratifying overall survival and risk of transformation to AML exist, such as the Revised International Prognostic Scoring System (IPSS-R). This score is based on the number and depth of cytopenias, marrow blasts and the presence of cytogenetic abnormalities. Conversion of all these elements into a numerical score generates five risk categories for MDS progression (IPSS-R)(Very Low, Low, Intermediate, High, Very High) [29]. A new Molecular International Prognosis Scoring System (M-IPSS) prognostic score was presented to the American Society of Hematology in 2021, integrating the mutational status of 38 gene loci that would allow for better risk discrimination as well as the reclassification of more than half of MDS patients into six risk strata (Very Low, Low, Moderate Low, Moderate High, High, Very High) [30]. The mutational status of UBA1 is not taken into account in the diagnosis and prognosis of MDS, related maybe to the recent description of this new syndrome.

Currently, the therapeutic management of MDS is of moderate efficacy and varies according to the age of patient, comorbidities and IPSS-R score. Thus, patients with low-risk MDS receive symptomatic treatment to correct cytopenias and avoid complications (transfusion, erythropoietin, etc.). On the other hand, patients under 65 years of age with high-risk MDS may have access to allogeneic HSC transplantation. Azacitidine is the reference treatment and is used in particular in high-risk MDS. Lenalidomide is an immunomodulator and is reserved for patients with an isolated 5q deletion who are better responders, provided there is no TP53 mutation.

The co-occurrence of MDS in patients with inflammatory disease has been identified in the literature as approximately 20% of MDS are associated with AID, with a pathophysiological mechanism between the two entities still undefined [31]. AID are heterogeneous (arthritis, connectivitis, vasculitis, neutrophilic dermatoses, etc.) and are related to a deregulation of the activation of innate and adaptive immune pathways [20]. Historically considered as hereditary diseases with Mendelian inheritance, AIDs can also be secondary to the acquisition of somatic mutations in HSC. The classification of AIDs is complex as some entities share features with autoimmune and autoinflammatory diseases. Currently, there is no pathophysiological data in the literature linking the development of MDS to AIDs and vice versa. Recently, the molecular landscape of a cohort including MDS patients with AID was reported with a higher frequency of mutations in the TET2, IDH1/2 and SRSF2 genes compared to a cohort of 319 MDS patients without AID [32].

It seems that the MDS heterogeneity of VEXAS patients is less important with essential MDS with uni- or multi-lineage dysplasia and rarely with a severe prognostic score [33]. The mutational profile of VEXAS-MDS also appears to be less complex, most often with no additional mutations found in high-throughput sequencing analysis. The few co-mutations described include DNMT3A, MLL, CSF1R, SF3B1, TET2, GNA11 and ZRSR2. It is not yet defined whether the UBA1 clone is the clonal initiating event of MDS or whether the myeloid neoplasm is the product of a highly inflammatory microenvironment responsible for clonal selection. Because VEXAS is a heterogeneous disease, it may be necessary to adjust therapies according to the hematologic phenotype. It would be expected that azacitidine, one of the current treatments for MDS and reported to be effective in MDS with AID, would also be effective in VEXAS patients with MDS. Despite encouraging results with azacitidine in VEXAS patients [8], follow-up of the efficacy of azacitidine in 4 VEXAS patients with MDS compared to 22 MAI MDS patients has not yet shown a conclusive result, suggesting a challenge for the therapeutic management of patients with UBA1 mutations [34].

## 4. Conclusions

To mitigate the high morbidity and mortality of VEXAS patients, it is important that they benefit from an early diagnosis based on a multidisciplinary consultation (rheumatologists, dermatologists, clinical hematologists and biologists) as well as from a regular clinical and molecular follow-up. The disappearance of the UBA1 clone on molecular sequencing after the introduction of a new therapeutic line would indeed be a good argument for its effectiveness. According to the published series, the prevalence of MDS in VEXAS patients is from 25 to 50% but these associated MDS appear to be different from classic MDS since the majority do not have additional molecular abnormalities, and their cytological presentation is less heterogeneous. The discovery of VEXAS syndrome also illustrates the power of molecular sequencing as a tool for identifying the role of mutations in somatic diseases, which is probably very underestimated in autoinflammatory and immune diseases. Using genomic rather than phenotypic data first to discover genes responsible for new diseases was a winning bet for Beck and his team, who were able to overcome clinical disparities between patients and then group them together. In general, the identification of VEXAS syndrome shows the importance of a more systematic integration of genetic data in the classification of somatic diseases. The multiplication of prospective follow-ups and functional studies will allow us to better elucidate the pathophysiological mechanism of VEXAS disease and the development of new therapeutics essential to the management of this disease with a poor prognosis.
